# Essential Roles for the Non-Canonical IκB Kinases in Linking Inflammation to Cancer, Obesity, and Diabetes

**DOI:** 10.3390/cells8020178

**Published:** 2019-02-19

**Authors:** Chong Hyun Shin, Doo-Sup Choi

**Affiliations:** Department of Molecular Pharmacology and Experimental Therapeutics, Mayo Clinic, Rochester, MN 55905, USA; Choids@mayo.edu

**Keywords:** TBK1, IKKε, inflammation, cancer, obesity, metabolic disease, diabetes

## Abstract

Non-canonical IκB kinases (IKKs) TBK1 and IKKε have essential roles as regulators of innate immunity and cancer. Recent work has also implicated these kinases in distinctively controlling glucose homeostasis and repressing adaptive thermogenic and mitochondrial biogenic response upon obesity-induced inflammation. Additionally, TBK1 and IKKε regulate pancreatic β-cell regeneration. In this review, we summarize current data on the functions and molecular mechanisms of TBK1 and IKKε in orchestrating inflammation to cancer, obesity, and diabetes.

## 1. Introduction

Nuclear factor κB (NF-κB) pathway plays a crucial role in multiple pathological conditions such as cancer, obesity and metabolic disease, and diabetes [1,2,3,4,5,6,7,8,9,10,11,12,13,14,15,16]. In particular, NF-κB activation is common in a wide range of tumors, suggesting that NF-κB serves as a bridge between inflammation and cancer. The NF-κB-dependent gene expression in obese adipose tissue and liver plays an important role in insulin resistance and type 2 diabetes. In the case of skeletal muscle, consisting of muscle fibers and connective and adipose tissues, previous studies suggest that activation of NF-κB primarily in the adipose tissue macrophages (ATMs) of skeletal muscle, not muscle fibers, contributes to development of insulin resistance in the context of obesity [17,18,19,20,21,22,23]. Moreover, cytokine-triggered NF-κB activation results in dysfunction and death of β-cells in type 1 diabetes.

NF-κB represents a family of inducible transcriptional factors that regulates a large array of genes involved in different processes of the inflammatory response [24]. In mammalian cells, the NF-κB family is composed of five structurally related members, including RelA (also named p65), RelB, c-Rel, NF-κB1 (also named p50), and NF-κB2 (also named p52) [25]. Distinct NF-κB complexes bind to a specific DNA element, κB enhancer, as various homo- and hetero-dimers [25]. In most cell types, NF-κB complexes are retained in the cytoplasm by a family of inhibitory proteins, including inhibitors of NF-κB (IκBs) and related proteins characterized by the presence of ankyrin repeats [26,27,28]. To date, IκBα is the most well studied member of the IκB family [26,27,28]. The activation of NF-κB involves two major signaling pathways, the canonical (or classical) and non-canonical (or alternative) pathways [26,29]. The canonical NF-κB pathway is typified by activation of the IκB kinase (IKK) complex for phosphorylation of IκBα at Serine 32 (Ser32) and Serine 36 (Ser36) [24,30]. IKK complex is composed of two catalytic subunits, IKKα and IKKβ, and a regulatory subunit named NF-κB essential modifier (NEMO or IKKγ). Different stimuli, such as cytokines, growth factors, mitogens, microbial components, and stress agents [31,32], can activate IKK complex. Phosphorylated IκBα is subject to ubiquitin-induced proteasomal degradation for rapid and transient nuclear translocation of canonical NF-κB members, predominantly p50-RelA and p50-c-Rel dimers [27,30,33]. In contrast to the canonical NF-κB pathway, the non-canonical pathway selectively responds to a specific group of stimuli [34,35]. In addition, activation of the non-canonical NF-κB pathway does not involve IκBα degradation but rather relies on processing of the NF-κB2 precursor protein p100 to p52 by NF-κB inducing kinase (NIK) and IKKα [26,36,37,38]. The non-canonical p52-RelB heterodimers have a higher affinity for distinct κB elements and regulate a distinct subset of NF-κB target genes [39].

**Figure 1:**
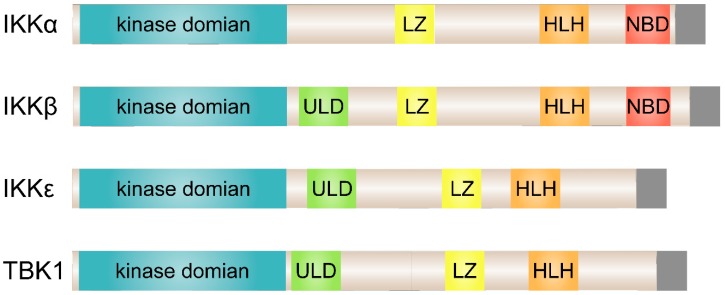
Structural comparison of the classical and non-canonical IκB kinases (IKKs). The kinase domain of IKKε exhibits 27% and 24% identity to IKKα and IKKβ, respectively, and TBK1 shares 49% identity and 65% similarity to IKKε. ULD, ubiquitin-like domain; LZ, leucine zipper; HLH, helix-loop-helix; NB, NEMO-binding domain.

Two IKK-related kinases, TANK (TRAF-associated NF-κB activator) binding kinase 1 (TBK1) and IKKε (also known as IKK-inducible or IKK-i), were discovered as the non-canonical IKKs [40,41,42]. TBK1 is ubiquitously expressed in all tissues, whereas IKKε expression is restricted to particular tissues, with highest levels detected in lymphoid tissues, peripheral blood lymphocytes, and the pancreas [41,43]. As shown in Figure 1, although TBK1 and IKKε have a similar domain composition as the canonical IKKs, they lack a NEMO-binding domain (NBD) and are dispensable for IκBα phosphorylation, indicating that they do not act as IκB kinases [44,45]. Accordingly, the primary function of TBK1 and IKKε is to activate type I interferon (IFN) genes (IFN-α and IFN-β) in innate immune cells [46,47]. Recent evidence suggests that tumor necrosis factor α (TNFα) induces TBK1 and IKKε, which play pivotal roles as mediators of obesity-induced systemic low-grade inflammation [10,11]. Furthermore, small molecule inhibitor driven suppression of the activity of these kinases enhanced regeneration of pancreatic β-cells in multiple species including zebrafish, mice, and humans [48].

## 2. TBK1 and IKKε in NF-κB Signaling

As identified activators of NF-κB, TBK1 and IKKε target multiple NF-κB members and effectors [40,43,49]. While TBK1 and IKKε phosphorylate IκBα, phosphorylation is efficient at only one of the two serine residues typically targeted on IκBα [41,43,49], RelA and c-Rel are other substrates for TBK1 and IKKε [50,51]. Independent of extracellular stimuli, TBK1 and IKKε phosphorylate RelA at Ser536 at a basal level. It may explain the low level of constitutive NF-κB activity in many cell types [50,51]. Phosphorylation of c-Rel is sufficient to dissociate c-Rel-IκBα complex and promote nuclear translocation of c-Rel [52].

TBK1 and IKKε phosphorylates distinct substrates in NF-κB pathway, thus, they may activate NF-κB through different mechanisms. Only TBK1 phosphorylates and activates IKKβ, functioning additionally as an IKK kinase [43]. On the other hand, in stimulated T cells, IKKε phosphorylates RelA at Ser468 [53]. Small interfering RNA driven downregulation of IKKε primarily prevented Ser468 phosphorylation without affecting inducible phosphorylation of Ser536. The Ser468 phosphorylated form of RelA occurred mainly in the nucleus, whereas Ser536 phosphorylated form predominantly in the cytosol, suggesting a function for transactivation [53]. IKKε also associates with p52 and its precursor p100 in a ternary complex with RelA following TNFα induction. This interaction facilitates transactivation of p52 dependent genes [54].

While TBK1 and IKKε are capable of regulating multiple NF-κB members and effectors, studies also showed that TBK1 and IKKε are not required for NF-κB activation. IκBα degradation or NF-κB-DNA binding in TBK1- or IKKε-deficient murine embryonal fibroblasts (MEF) was unaltered after stimulation with TNFα, interleukin (IL)-1β, lipopolysaccharide (LPS), and polyI:C, respectively [55,56]. Essentially, it appears that TBK1 and IKKε do not generally target NF-κB signaling and the role of these kinases in NF-κB activation is highly dependent on cellular and signal-induced contexts [57,58,59].

In line with these findings, mice lacking either *Tbk1* or *Ikbke* exhibit distinct phenotypes. *Tbk1*-deficient animals are phenotypically similar to NEMO-, IKKβ-, and RelA-deficient mice with embryonic lethality at E14.5 due to extensive fetal liver degeneration [44]. By contrast, *Ikbke*-deficient animals are viable but are important for the activation of IFN-β and IFN-inducible genes [55]. NF-κB activation in *Tbk1* and/or *Ikbke* knockout models is overall normal, apart from minimal defects in the induction of select NF-κB target genes.

## 3. TBK1 and IKKε in Interferon Signal Transduction

**Figure 2:**
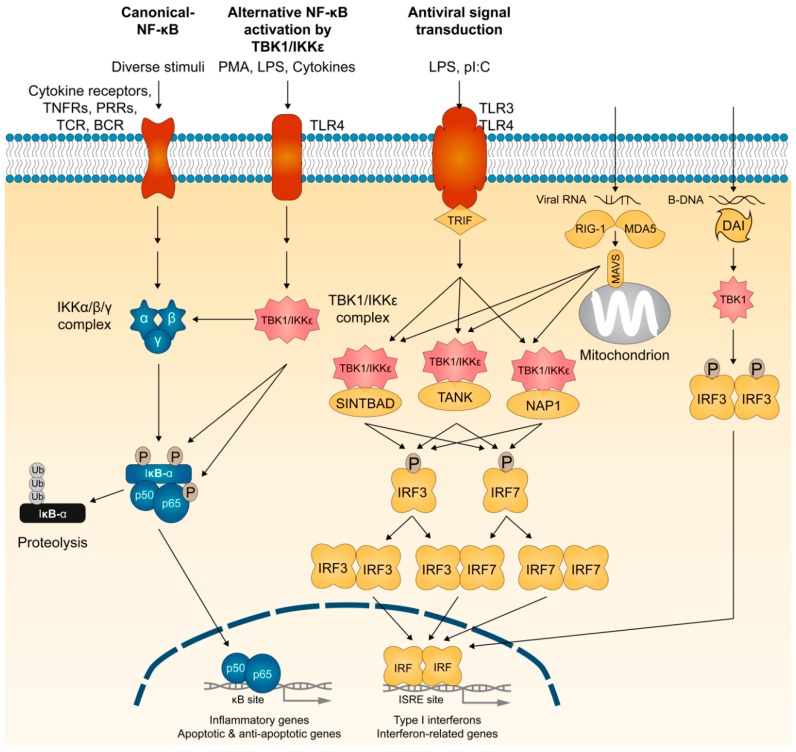
The membrane and cytosolic TBK1- and IKKε-dependent signaling pathways. Viral or bacterial products trigger signaling pathways through the membrane-bound Toll-like receptors (TLRs) or the cytosolic RNA and DNA sensors. Both signaling cascades rely on the coordinated activation of transcription factors, such as interferon (IFN) regulatory factors (IRFs). IRF3 and IRF7 activation in the cytoplasm occurs directly through their C-terminal phosphorylation by TBK1 and IKKε, which promote IRF3 and IRF7 homo- and hetero-dimerization and their subsequent nuclear import. TANK, NAP1, and SINTBAD play essential roles in the assembly of TBK1 and IKKε kinase complexes. IRF3 and IRF7 activation is also triggered when cytosolic receptors sense intracellular nucleic acids (RNA from viruses or DNA from viruses or damaged cells). RNA from viruses triggers the activation of the cytosolic receptors RIG-I and MDA-5, and their subsequent binding to mitochondrial adaptor MAVS. Cytosolic DNA is detected by the system called DAI (DNA-dependent activator of IRFs). As originally identified as activators of NF-κB, TBK1 and IKKε also target multiple NF-κB members and effectors. TNFR, TNF receptor; PRR, pattern recognition receptor; TCR, T-cell receptor; BCR, B-cell receptor; PMA, phorbol myristate acetate; LPS, lipopolysaccharide; pI:C, polyinosinic:polycytidylic.

Innate immune cells express pattern recognition receptors (PRRs) that detect pathogen-associated molecular patterns (PAMPs) presenting on bacteria and viruses [60,61]. As a consequence, induction of genes encoding the type I IFNs (IFN-α and IFN-β), proinflammatory cytokines, and chemokines occurs [62,63]. There are two broad classes of PRRs: (1) membrane-bound Toll-like receptors (TLRs) that utilize adaptor proteins TRIF (TIR-domain-containing adaptor protein inducing IFN-β) or MyD88 (myeloid differentiation primary-response protein 88) and (2) cytosolic pattern recognition receptors (PRRs) including RIG-I (retinoic acid-inducible gene-I)-like receptors, NOD (nucleotide-binding oligomerization domain)-like receptors (NLRs), and cytosolic DNA sensors [60,64,65]. Engagement of these receptors activates the NF-κB and IFN regulatory factors (IRFs). Although simultaneous activation of both the NF-κB and IRF families of transcription factors takes place, the induction of proinflammatory cytokines requires NF-κB, whereas type I IFN gene induction mainly relies on IRF activation. In contrast to NF-κB activation, which relies on the degradation of IκBα and subsequent release of NF-κB proteins, IRF3 and IRF7 activation in the cytoplasm occurs directly through their C-terminal phosphorylation at multiple serine and threonine residues by TBK1 and IKKε [66,67,68,69,70]. These modifications promote IRF3 and IRF7 dimerization and nuclear translocation, as illustrated in Figure 2.

Several scaffolding effectors regulate the kinase activities of TBK1 and IKKε. Whereas NEMO assembles some but not all IKK complexes, studies provide strong experimental evidence for a role of TANK (also called TRAF-interacting protein (I-TRAF)) [71,72,73,74], NAK-associated protein (NAP1) [51,75], and similar to NAP1 TBK1 adaptor (SINTBAD) [76] in the assembly of TBK1 and IKKε kinase complexes that phosphorylate IRF3 and IRF7, and promote type I IFN gene induction (Figure 2). In addition, viral RNA is detected by cytosolic PRRs such as RIG-I and MDA-5 (melanoma differentiation-associated gene 5) [77,78]. Mitochondrial antiviral signaling adaptor MAVS (also known as IPS-1, VISA, or Cardif) relays signals from RIG-I and MDA-5 to TBK1 and IKKε for phosphorylation of IRF3 and IRF7 [79,80,81,82]. Cytosolic DNA-sensing system called DAI (DNA-dependent activator of IRFs), also known as DLM-1 or Z-DNA binding protein 1 (ZBP1), is assembled TBK1 and IRF3 for IFN-β induction [83]. IFN-β also activates a TLR-independent pathway by stimulating IKKε phosphorylation of Ser708 on STAT1 (signal transducer and activator of transcription 1) to have a more stable STAT1-STAT2-IRF9 interaction for binding of ISGF3 complex to ISREs (interferon-stimulated response elements), which serves as the transcriptional machinery important for activating a subset of interferon response genes [84]. Thus, TBK1 and IKKε form several protein complexes that share a role in activating interferon responses required to induce the anti-viral responses.

## 4. TBK1 and IKKε in Cancer

Increasing evidence has revealed that both TBK1 and IKKε participate in signaling pathways that impact cell transformation and tumor progression. TBK1 plays an important role in activating anti-apoptotic pathways in cells mutated for the proto-oncogene KRAS. A variety of cancers, including pancreatic, colorectal, and non-small cell lung cancer, have KRAS mutations at a high frequency [85]. RalB, one of the monomeric RalGTPases activated by Ral-GEF (Ras-like- guanine nucleotide exchange factor), functions to mediate TBK1 activation in tumorigenic transformation and suppress apoptotic checkpoint activation [86]. RalB and its effector protein Sec5, a component of the octameric exocyst complex (Sec3, Sec5, Sec6, Sec8, Sec10, Sec15, Exo70, and Exo84), directly recruits and activates TBK1 [86]. Expression of oncogenic alleles of KRAS induced cell death in TBK1-deficient murine embryonic fibroblasts, suggesting that RalB-Sec5-TBK1 controls a cell-autonomous host defense signaling pathway that inhibits tumor cell apoptosis [86]. In contrast, upon stimulation with dsRNA or Sendai virus, RalB-Sec5-TBK1 pathway activates TLR without affecting the survival of non-tumorigenic epithelial cells [86]. Thus, in tumor cells the RalB-Sec5-TBK1 pathway inhibits apoptosis, whereas in non-tumorigenic cells, it stimulates an innate immune response. In addition, TBK1 has an oncogenic role in melanoma, non-small cell lung cancer (NSCLC), HTLV-1 (human T-cell leukemia virus type 1), and breast cancer [87,88,89,90]. Accordingly, inhibitors of TBK1/IKKε induced apoptosis in a subset of BRAF inhibitor (BRAFi)-resistant tumors [87]. Moreover, a subset of NSCLC cells exhibited sensitivity to TBK1 inhibition by blunting Akt and mTORC1 (mechanistic target of rapamycin complex 1) signaling [88].

NF-κB pathway regulates IKKε in multiple human cancers [91,92]. NF-κB pathway functions in a cell type-specific manner. It activates survival genes within cancer cells and inflammation-promoting genes in components of the tumor microenvironment. One of the specific substrates for IKKε involved in cell transformation is the tumor suppressor CYLD [93]. CYLD is a deubiquitinating enzyme (DUB) that removes Lys63-linked ubiquitin chains in several NF-κB regulators, including TRAF2 and TRAF6 as well as NEMO, thus acting as a negative regulator of NF-κB signaling [94,95,96]. Overexpression of IKKε alone is sufficient to drive transformation of NIH-3T3 cells by phosphorylating CYLD at Ser418 and decreasing its deubiquitinase activity [93]. In breast carcinomas and breast cancer cell lines, elevation of the levels of serine/threonine kinase CK2 (casein kinase 2) and amplification/overexpression of IKKε take place [97]. CK2 phosphorylates C-terminal PEST (Ser283, Ser289, Thr291, and Ser293) domain of IκBα, thereby, affecting the turnover of IκBα and increasing NF-κB activity. Ectopic expression of CK2 subunits enhanced IKKε levels in mammary tumors. Conversely, suppression of CK2 in breast cancer cell lines reduced endogenous IKKε levels. In line with these data, expression of the kinase-inactive form of IKKε in breast cancer cells reduced levels of two NF-κB target genes, Cyclin D1 and RelB [97]. Treatment of CYT387, an inhibitor of TBK1/IKKε and JAK signaling, also impaired the viability of multiple different triple-negative breast cancer (TNBC) cell lines where IKKε is aberrantly overexpressed [98]. The JAK inhibitor ruxolitinib alone did not impede proliferation of TNBC cells. In glioma cell lines and in human glioma tissues, levels of IKKε mRNA and proteins levels increase. Overexpression of IKKε in glioma cells displayed decreased activity of caspase 3 but increased levels of Bcl-2, an anti-apoptotic protein, and NF-κB transactivation activity by increasing nuclear translocation of RelA and p50 proteins [99], while silencing IKKε decreased translocation [99].

IKKε expression is elevated in pancreatic ductal adenocarcinomas (PDACs). Accordingly, the survival time of patients with augmented levels of IKKε is poor [100]. Moreover, IKKε is a direct target of an effector of Hedgehog (Hh) signaling pathway, GLI1, and modulates GLI1 activity by controlling its nuclear localization in KRAS-positive pancreatic models [101]. Comprehensive mechanistic study showed that IKKε promotes the reactivation of AKT post-inhibition of mTOR in PDAC cells [101]. Ovarian cancer patients with increased IKKε levels also had lower survival rates and a poor prognosis [102]. Cells overexpressing IKKε were resistant to cisplatin treatment, while knockdown of IKKε overcame cisplatin resistance. Although not being the sole mechanism of promoting ovarian cancer metastasis, IKKε expression was increased in metastatic ovarian cancers and showed uniformly low expression in primary sites of ovarian cancer. IKKε depletion in metastatic ovarian cancer cell lines decreased growth, adhesion, and invasion, while overexpression of IKKε in a less invasive ovarian cancer cell line increased metastasis in vivo [103]. Inflammatory cytokine interleukin 6 (IL-6) serves as a growth factor in prostate cancer cells and is elevated in serum as well as in cancer tissue of prostate cancer patients [104,105]. In prostate cancer cell-based and xenograft models, IKKε promoted proliferation and tumor growth along with IL-6 expression in a manner dependent on the nuclear accumulation of the transcription factor C/EBP-β, which regulates genes involved in metastasis and survival of prostate cancer cells [106].

## 5. TBK1 and IKKε in Obesity

Obesity is associated with chronic low-grade inflammation, which develops insulin resistance and type 2 diabetes [107,108,109,110,111]. While the precise molecular links between inflammation and disrupted glucose homeostasis are not completely understood, NF-κB signaling is involved in inflammatory signaling downstream of the diverse initiators of adipocyte inflammation, including gut-derived antigens, dietary or endogenous lipids, and hypoxia [111,112,113]. Consequently, disruption of NF-κB signaling via targeted knockout of the canonical IκB kinase IKKβ gene or pharmacological inhibition of this pathway can restore insulin sensitivity in obese states [8,19,21,114,115].

Unlike canonical IKKs, activation of NF-κB by high fat diet (HFD) induces TBK1 and IKKε expression in metabolic tissues including fat and liver, with the most profound increase in adipocytes and ATMs (adipose tissue macrophages) [10,11]. IKKε knockout mutant mice gained far less weight than wild-type mice when fed a HFD due to enhanced oxygen consumption, leading to increase heat generation (thermogenesis) and core body temperature [10]. Expression of the uncoupling protein UCP1, which uncouples mitochondrial oxidative phosphorylation and augments thermogenesis, was markedly increased in white adipose tissue (WAT) in IKKε-deficient mice. These studies suggest that IKKε regulates thermogenesis in response to dietary fat consumption by hindering UCP-1-mediated uncoupled oxidative phosphorylation during mitochondrial respiration. In addition, mice lacking IKKε also display pronounced improvements in glucose and lipid homeostasis, amelioration of insulin resistance, and decreased activation of chronic, but not acute, inflammatory pathways [10]. Expression of wild-type IKKε in cultured adipocytes suppressed glucose transport activated by insulin, whereas kinase-defective IKKε displayed a minimal effect. Consistent with IKKε’s ability to facilitate chronic inflammation, expression of wild-type IKKε enhanced the levels of proinflammatory genes in hepatocytes. Thus, some of the effects of IKKε deletion are likely to be exerted in a cell- or tissue-autonomous manner although decreased adiposity itself confers insulin sensitivity and reduces inflammation.

A library screen of 150,000 chemical compounds identified amlexanox, a high-affinity pharmacological inhibitor of IKKε [11]. In vitro studies revealed that amlexanox blocks TBK1 as well [11]. Daily oral administration of amlexanox prevented HFD-induced weight gain in mice over a 12-week period [11]. Moreover, amlexanox treatment in two different mouse models of obesity (HFD- induced and leptin-resistant *ob/ob* mice) resulted in improved insulin sensitivity, attenuated hepatic steatosis, reduced adipose tissue inflammation, and promoted energy expenditure in adipose tissue through increased thermogenesis [11]. Intriguingly, suppression of TBK1 and IKKε in adipocytes enhanced some aspects of the initial NF-κB response to cytokines or LPS, potentially due to the lack of feedback inhibition that is a consequence of elevated expression of TBK1 and IKKε. These results indicate that TBK1 and IKKε function as “counter-inflammatory” kinases that maintain the low-grade, chronic inflammation in obesity by preventing its resolution while sustaining energy conservation. Thus, these non-canonical kinases are not directly proinflammatory and do not act as IκB kinases.

In a placebo-controlled study of 42 obese patients with type 2 diabetes (T2D) and nonalcoholic fatty liver disease, amlexanox treatment significantly reduced Hemoglobin A1c and fructosamine [116]. A subset of drug responders also exhibited improvements in insulin sensitivity and hepatic steatosis, following a transient increase in serum IL-6 levels. This subgroup was characterized by higher inflammatory gene expression from biopsied subcutaneous fat and greater serum C-reactive protein (CRP) levels than non-responders at baseline. They also exhibited a unique pattern of thermogenic gene expression changes, including UCP1, DIO2, and FGF21, in subcutaneous white adipose tissue in response to amlexanox, consistent with the browning of the adipose tissue observed in mice.

**Figure 3:**
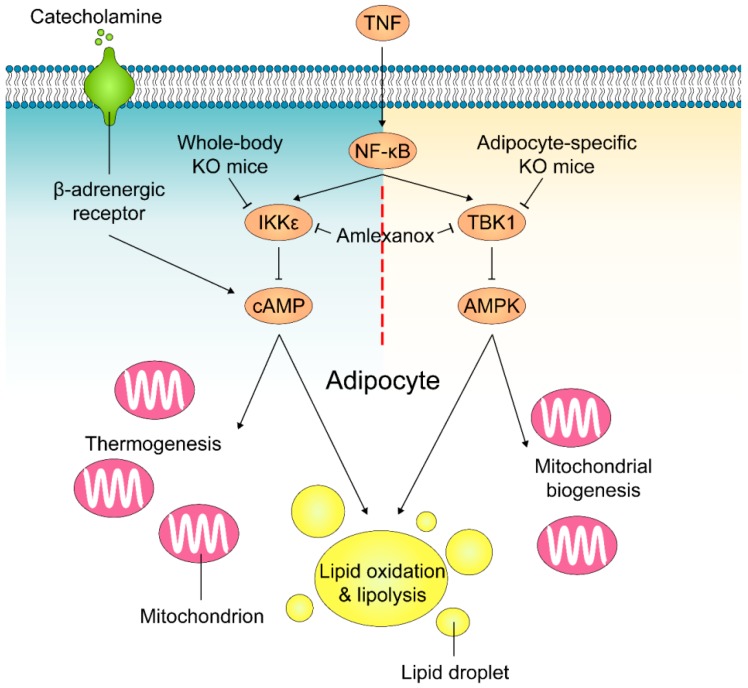
Mechanisms of how TBK1 and IKKε regulate lipolysis and energy expenditure in adipocytes. Obesity-accompanied TNFα stimulates NF-κB activity and induces TBK1 and IKKε expression in adipocytes. IKKε phosphorylates and activates PDE3B to decrease intracellular cAMP levels and induce catecholamine resistance, resulting in a reduction in lipolysis and thermogenesis in response to sympathetic activation in adipose tissue. In parallel, NF-κB-induced TBK1 decreases lipid oxidation and significantly reduces mitochondrial biogenesis by inhibiting AMPK activity in adipocytes. Amlexanox inhibits the activity of both TBK1 and IKKε.

Adipose tissue becomes less sensitive to catecholamines, such as adrenaline, in states of obesity. This reduced sensitivity in turn decreases energy expenditure. As plausible mechanisms of how TBK1 and IKKε preserves energy storage, Mowers et al. showed that elevated levels of these two enzymes reduced the ability of β-adrenergic receptors in the fat cells of obese mice to respond to catecholamines, resulting in lower levels of cyclic AMP (cAMP) (Figure 3) [117]. Upon increased expression in the obese state, TBK1 and IKKε phosphorylate and increase the activity of cAMP hydrolyzing enzyme phosphodiesterase 3B (PDE3B) [118], decreasing cAMP-dependent phosphorylation of proteins in response to sympathetic activation. These proteins include hormone sensitive lipase (HSL) and perilipin that are responsible for β-adrenergic-stimulated lipolysis, and other proteins, such as p38, that regulates expression of UCP1. Accordingly, the reduced sensitivity to β-adrenergic activation attenuates lipolysis and fatty acid oxidation as well as adaptive thermogenesis.

A euglycemic–hyperinsulinemic clamp revealed that suppression of hepatic glucose production primarily attributes to the insulin-sensitizing effects of amlexanox [11]. RNA sequencing analysis of hepatic gene expression a few hours after in vivo amlexanox treatment identified over 1700 differentially expressed genes [119]. The top two most enriched pathways were the adipokine signaling pathway and the JAK/STAT signaling pathway. Inhibition of TBK1 and IKKε by amlexanox stimulated the secretion of cytokine IL-6, which is upstream of the JAK/STAT pathway, from adipocytes as well as preadipocytes in the subcutaneous adipose tissue via a cAMP/p38-dependent pathway. The resulting increase in serum IL-6 is responsible for the activation of hepatic STAT3, which suppresses expression of *G6pc* to reduce hepatic glucose output [120,121,122].

Adipocyte-specific TBK1 knockout (ATKO) attenuated HFD-induced obesity by increasing energy expenditure [123]. Surprisingly, while amlexanox treatment improved catecholamine sensitivity in adipose tissue and reduced insulin resistance/adipose tissue inflammation [11], ATKO exaggerated HFD-induced glucose intolerance and insulin resistance due to enhanced adipose inflammation and macrophage infiltration [123]. Detailed biochemical and functional studies revealed that TBK1 directly inhibits AMP-activated protein kinase (AMPK) [124,125] to repress respiration and increase energy storage (Figure 3). Conversely, activation of AMPK under catabolic conditions can increase TBK1 activity through phosphorylation by AMPK’s downstream target ULK1 (Unc-51-like autophagy-activating kinase 1) [126]. Furthermore, TBK1 suppresses inflammation by phosphorylating and inducing the degradation of the IKK kinase NIK, thus, attenuates NF-κB activity and mediates the negative impact of AMPK activity on NF-κB activation. This shows that TBK1 plays a unique role in mediating bidirectional crosstalk between energy sensing and inflammatory signaling pathways in both over- and under-nutrition.

## 6. TBK1 and IKKε in Diabetes

Diabetes is characterized by impaired glucose homeostasis resulting from insufficiency or functional failure of insulin-producing β-cells, alone or in association with insulin resistance [127,128,129,130,131]. As both metabolic factors and immune components promote progression of diabetes [127,128,130,132,133,134,135], coupling expansion and protection of residual functional β-cells is critical in remedying diabetes.

Islet inflammation plays a key role in decreasing functional β-cell mass in both type 1 diabetes (T1D) and type 2 diabetes (T2D) [136,137]. In T1D, β-cells are the target of an autoimmune assault. In obesity-induced insulin resistance and T2D, chronic low-grade inflammation and activation of the immune system are primary etiological factors. Inflammation occurs in the insulin-sensitive tissues, such as adipose tissue, liver, skeletal muscle, and pancreas, which results in β-cell dysfunction and apoptosis [138].

Because both T1D and T2D eventually lead to β-cell loss, research has focused on developing β-cell replacement strategies to compensate for insulin deficiency, including in vitro differentiation of human pluripotent stem cells (hPSCs) toward β-cells and in vivo regeneration approaches aimed at replenishing β-cell mass [139,140]. β-cell regeneration can be promoted by either increasing residual β-cell proliferation or stimulating neogenesis of new β-cells from non-β-cells [141,142,143,144]. Non-β-cells include progenitors residing in the extra- and/or intra-pancreatic ductal structures and other mature cell types, including glucagon-expressing α-cells or digestive enzyme-secreting acinar cells.

**Figure 4:**
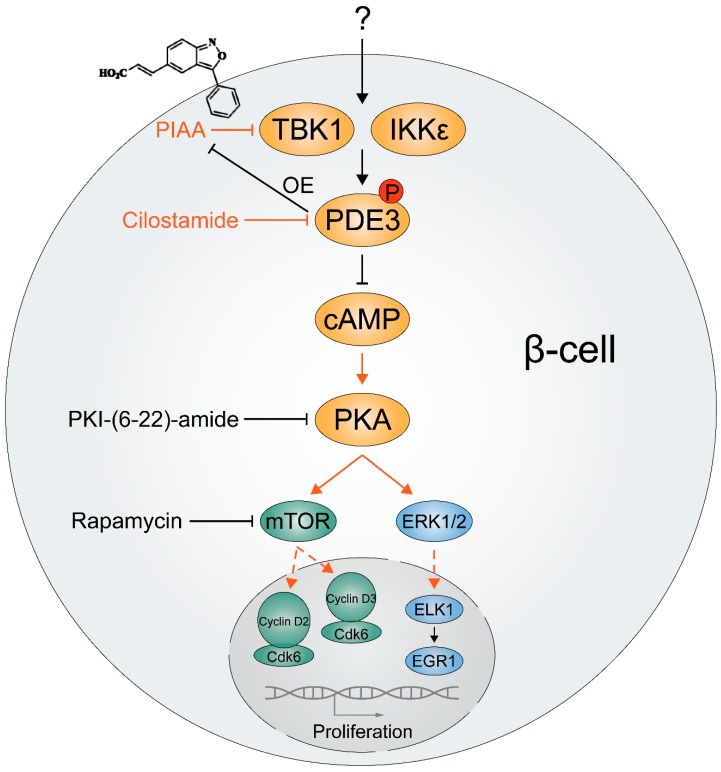
Plausible mechanisms of how TBK1/IKKε control proliferation of β-cells. A cinnamic acid derivative (E)-3-(3-phenylbenzo[c]isoxazol-5-yl)acrylic acid (PIAA), a novel small molecule inhibitor of TBK1/IKKε showing effective β-cell regeneration potency, stimulates β-cell-specific proliferation. Genetic overexpression of PDE3, β-cell-enriched cyclic AMP (cAMP) hydrolyzing enzyme, and pharmacological inhibition of cAMP-dependent protein kinase A (PKA) and mechanistic target of rapamycin (mTOR) blunted PIAA-mediated β-cell regeneration, implicating that TBK1/IKKε suppress cAMP-PKA-mTOR signaling axis via PDE3 to reduce functionally relevant β-cells. As key cell cycle molecules are constrained to the cytoplasm in quiescent human β-cells and potentially also rodent β-cells, it is plausible that TBK1/IKKε inhibition drives proliferation of β-cells by translocating them including mTOR-regulated cyclins D2 and D3 into the nucleus. Additionally, phosphorylation of ERK1/2 was induced by PIAA, suggesting an involvement of the cAMP-PKA-ERK1/2 signaling axis in β-cell proliferation. OE, overexpression.

A recent study unveiled a novel function of TBK1 and IKKε in regulating β-cell regeneration [48]. Given the slow rate of β-cell regeneration in adult humans [145,146], Xu and colleagues used a transgenic zebrafish model of T1D and performed a chemical–genetic screen to identify additional small molecule enhancers of β-cell regeneration [48]. They identified inhibitors of TBK1 and IKKε as enhancers of β-cell regeneration. The most potent β-cell regeneration enhancer was a cinnamic acid derivative (E)-3-(3-phenylbenzo[c]isoxazol-5-yl)acrylic acid (PIAA), acting through the cAMP-dependent protein kinase A (PKA) (Figure 4). PIAA stimulated β-cell-specific proliferation by increasing levels of cyclic AMP (cAMP) and activity of mTOR, a serine/threonine protein kinase essential for cell growth and metabolism [147]. A combination of PIAA and cilostamide, an inhibitor of β-cell-enriched cAMP hydrolyzing enzyme phosphodiesterase (PDE) 3 [148], enhanced β-cell proliferation, whereas overexpression of PDE3 blunted the mitogenic effect of PIAA in diabetic zebrafish. PIAA augmented proliferation of INS-1β-cells and β-cells in mammalian islets, including human islets, with elevation in cAMP levels and insulin secretion. PIAA improved glycemic control in streptozotocin (STZ)-induced diabetic mice with increases in β-cell proliferation, β-cell area, and insulin content in the pancreas. Thus, TBK1/IKKε suppression plays an evolutionarily conserved and critical role in expanding functional β-cell mass.

For a needed supply of energy and macromolecules, tumor cells maintain rapid growth by switching to glycolysis [149]. Increased expression of glucose transporters controls elevated glucose uptake in cancer [149]. Upon activation of RalA, TBK1 phosphorylates the exocyst protein Exo84, leading to translocation of the GLUT4 glucose transporter to the cell membrane [150]. TBK1 can phosphorylate the insulin receptor (Ser994) to block the activity of the receptor, potentially leading to insulin resistance [151]. Along a different line, AMPK was shown to phosphorylate and stabilize the tumor suppressor TET2 (tet methylcytosine dioxygenase 2) [152]. In this study, increased glucose blocked AMPK activity, resulting in destabilization of TET2 and reduced 5-hydroxymethylcytosine, which regulates DNA methylation status. Thus, the ability of TBK1 to negatively regulate AMPK can be critical for reducing TET2 and associated epigenetic changes during the progression from pre-diabetes, overt diabetes, and cancer.

## 7. Conclusions and Future Perspectives

TBK1 and IKKε are essential for linking inflammation to a number of pathological conditions, including cancer, obesity, and diabetes. NF-κB effectors contribute to tumorigenesis in cell autonomous and non-cell autonomous manners. In addition, TBK1 and IKKε are induced by cytokines and closely associated with a decrease in energy expenditure. Recently, inhibitors of TBK1 and IKKε were shown to augment β-cell regeneration in animal models of diabetes and β-cells in human islets.

One of the most devastating complications of obesity is T2D. Up to ~95% of the diabetic people worldwide suffer from T2D. Most patients with T2D are obese or overweight, and numerous longitudinal studies link obesity with insulin resistance, defective insulin secretion, and disruption of other aspects of energy homeostasis. T2D is characterized by a decline in β-cell function, reduced β-cell mass, and insulin resistance, which is a forerunner of diabetes and culprit of β-cell exhaustion. Accordingly, treatment strategies for T2D aim to improve insulin sensitivity and restore β-cell function/mass. In this regard, modulating the activity of TBK1 and IKKε can be one of the key strategies to achieve this goal. In line with previous studies suggesting that modulation of cAMP levels via GPCR in β-cells is essential for β-cell replication, survival, and insulin secretion [153,154,155], Xu et al. provided a compelling evidence that inhibition of TBK1/IKKε enhances selective β-cell proliferation by increasing cAMP levels via PDE3. α_2_-adrenergic receptor antagonist mirtazapine and several PDE inhibitors including a PDE3 inhibitor cilostamide have displayed their potency to stimulate β-cell replication in a cAMP-dependent manner [153]. *Pde3b* knockout (KO) mice also exhibit enhanced insulin secretion [156]. However, *Pde3b* KO mice fail to suppress hepatic glucose production and display insulin resistance with a number of cAMP-signal transduction components being altered in *Pde3b*-deficient livers [156]. On the contrary, genetic deletion of IKKε and pharmacological inhibition of TBK1/IKKε improved insulin sensitivity through the inhibition of hepatic glucose production with reduction of PDE3B activity and increase of cAMP levels in adipocytes, not in livers, in obese mice [10,11]. Thus, modulation of PDE3 activity and cAMP levels resulting from suppression of TBK1/IKKε will lead to an increase in the number of functionally adequate β-cells with direct or indirect improvement of insulin sensitivity.

It is important to note that despite high sequence homology with comparable phosphorylation profiling of substrate(s) [58], TBK1 and IKKε present some difference. Adipose-specific genetic ablation of TBK1 attenuates diet-induced obesity with exaggeration in glucose intolerance/insulin resistance [123], whereas genetic deletion of IKKε increases energy expenditure with improvement in insulin sensitivity on a high fat diet [10]. As IKKε has no effect on AMPK phosphorylation, IKKε may phosphorylate and activate PDE3B to induce catecholamine resistance, whereas TBK1 inhibits AMPK activity to reduce catabolism via this pathway. In addition, inhibition of IKKε improves glucose homeostasis and inflammation, whereas TBK1 mediates the anti-inflammatory function of AMPK via negatively regulating NF-κB activation. Thus, further comprehensive molecular dissection and elucidation of TBK1- and/or IKKε-controlled signaling networks involved in obesity and metabolic disease, diabetes, and cancer will open up new avenues of therapies for balancing energy and glucose homeostasis, and preventing subsequent tumor progression.

While there are several specific small molecule inhibitors of TBK1 and IKKε, it is critical to consider minimizing toxic side effects upon synthesis of new inhibitors of TBK1/IKKε. Amlexanox blocks activity of TBK1 and IKKε with a half maximal inhibitory concentration (IC50) of approximately 1–2 μM. Biologically less characterized azabenzimidazole (AZ) derivatives 5c and 5e have IC50 of 0.032 μM and 0.102 μM (AZ-5c) as well as 0.038 μM and 0.204 μM (AZ-5e) against TBK1 and IKKε, respectively [157], compared to that of 0.4 μM and 1.07 μM (PIAA). Another AZ derivative AZ13102909 has an IC50 of 0.005 μM against TBK1, promoting apoptosis in melanoma cells [158]. It is noteworthy that AZ-5c and AZ-5e demonstrated significant toxicity when testing in the β-cell ablated zebrafish at nanomolar range (C.H. S., unpublished observation). Amlexanox is proven to be safe with a long history of use in patients having asthma and allergic rhinitis in Japan and aphthous ulcers in the US [159,160]. In a clinical trial of amlexanox for 42 patients with obesity and T2D or nonalcoholic fatty liver disease (NCT01975935), a subset of patients responded with a reduction in blood glucose [116]. While designing and validating analogues of amlexanox with more potent TBK1/IKKε inhibition activities and minimal toxicity are in progress [161,162], PIAA exhibited higher potency than amlexanox in β-cell regeneration in diabetic zebrafish with minimal deleterious effects [48]. Thus, further creation of new molecular structures with potent TBK1 and/or IKKε inhibition activities and minimal toxicity using the PIAA as a scaffold will allow us to develop legitimate strategies for maintaining energy and glucose homeostasis, and impeding subsequent tumor progression. In addition, proteolysis-targeting chimera (PROTAC) has emerged as a technology that can target a protein of interest for degradation. PROTACs contain one moiety that binds an E3 ligase linked with another moiety that binds to the target protein, resulting in ubiquitination and subsequent degradation of the target. Recently, a PROTAC directed to TBK1 was shown to specifically degrade TBK1 in cells while not affecting the IKKε [163]. Thus, utilization of a TBK1 PROTAC could functionally dissect roles of TBK1 from those of IKKε, which may be a more effective treatment method.

Last but not least, it is essential to consider that TBK1 and IKKε have critical roles as regulators of innate immunity by regulating multiple NF-κB members/effectors and IRFs, including IRF3 and IRF7, for induction of type I IFN genes. Consistent with its role in the innate immune response, TBK1 also promotes autophagy for cellular homeostasis and cytoprotection. TBK1 phosphorylates autophagy receptors and increases their binding affinity to ubiquitin chains that mark cargos, including ubiquitinated mitochondria and ubiquitin-coated intracellular bacteria, for delivery to autophagosomes [164,165,166,167,168]. In addition, recent study suggests that TBK1 represses RIPK1 (receptor-interacting serine/threonine-protein kinase 1)-dependent apoptosis and inflammation downstream of TNFR1 (tumor necrosis factor receptor 1). TBK1 heterozygosity in mice bestows genetic susceptibility to amyotrophic lateral sclerosis (ALS)/frontotemporal dementia (FTD) [169], consistent with a high disease penetrance of TBK1 loss-of-function variants in mutation carriers in sporadic ALS/FTD [170]. Accordingly, non-selective and complete inhibition of these non-canonical IKKs might also lead to undesirable side effects by interfering with their function in the immune system, leading to increased susceptibility to infections and inflammatory disorders. In this context, it will be informative to modulate the expression/activity of TBK1 and IKKε in distinct cell types or tissues by means of conditional knock-out/knock-in/transgenic animal models and predict toxic effects of suppression of TBK1 and IKKε. Moreover, it will be important to further determine the relative contribution of TBK1 and IKKε in different pathophysiological processes. Eventually, all these studies can lead to the development of novel therapeutic agents that selectively repress disease-related TBK1 and IKKε activity with basal activity unaffected.
